# Deficiency of regulatory B cells in a house dust mite model of asthma

**DOI:** 10.1186/2045-7022-3-S1-P21

**Published:** 2013-05-03

**Authors:** Faouzi Braza, Julie Chesne, Guillaume Mahay, Marie-Aude Cheminant, David Lair, Antoine Magnan, Sophie Brouard

**Affiliations:** 1 Université de Nantes, UMR_S1084, UMR_S 1087, Institut du Thorax, France; 2 Université de Nantes, UMR_S 1087, Hopital Laënec, Institut du Thorax, L’institut du Thorax, France; 3 UMR_S 1087, Institut du Thorax, France; 4 UMR_S1084, CHU Hotel Dieu, ITUN, France

## Background

Asthma is a chronic disorder leading to bronchial obstruction in response to inhaled allergen. It is associated with immune deregulation with specific expansion of Th2 and Th17 CD4+ T cells. Both T cell populations support B cells response by stimulating their proliferation, survival and IgE secretion. B cells are described for their effector functions but recent reports have described their regulatory role in autoimmune and inflammatory disorders. However, definitive identification has been challenging because regulatory B cells (Breg) are rare, do not have a specific marker, and express detectable IL-10 or TGF-B only upon ex vivo stimulation. In asthma models local inhalation tolerance and helminth infection induce the generation of regulatory B cells. But no physiological role of this population in the development of asthma has been described yet.

## Methods

Mice were sensitized on days 0, 7, 14 and 21 by percutaneous administration of HDM onto the ears. Intra-nasal challenges were performed on day 27 and 34 with 250 µg HDM. One day after each challenge, we realized by flow cytometry a complete B cell phenotyping in spleen and lungs. Splenocytes and lung cells were isolated and stimulated ex vivo with LPS and PMA, ionomycin to induce IL-10 secretion by B cells.

## Results

No differential frequency was observed for all B cell populations in the spleen of HDM allergic mice, suggesting a normal B cell development. In contrast, HDM allergic mice exhibit a strong infiltration of CD19+ B cells in lungs and broncho-alveolar lavage after the second challenge. We found an increase of CD19 IgDhi IgMlow B2 mature and CD19 IgD- IgM- switched memory B cells in the lung of HDM allergic compared to control mice. We looked at CD19+ IL-10+ CD1dhi CD5+ CD21+ CD24hi IgMhi B cell population that has been shown to display regulatory properties in other situations. Whereas this population is present in spleen and lungs of HDM allergic mice, it produce less IL-10 than control after the first (vs control, p<0.001) and the second challenge (vs control, p<0.05) both in lung and spleen (vs control, p<0.05).

## Conclusion

These results suggest a potential defect of B cell regulation in asthma. Future investigations will focus on their regulatory capacities *in vitro* and *in vivo*.

